# Prospective, longitudinal study to isolate the impacts of marijuana use on neurocognitive functioning in adolescents

**DOI:** 10.3389/fpsyt.2023.1048791

**Published:** 2023-05-15

**Authors:** Wen Ren, Diana Fishbein

**Affiliations:** ^1^Department of Human Development and Family Studies, The Pennsylvania State University, University Park, PA, United States; ^2^Frank Porter Graham Child Development Institute, University of North Carolina-Chapel Hill, Chapel Hill, NC, United States

**Keywords:** adolescence, marijuana use, neurocognitive functioning, emotion recognition, longitudinal, substance-naïve baseline, legalization

## Abstract

**Introduction:**

Policies to legalize possession and use of marijuana have been increasingly supported across the United States. Although there are restrictions on use in minors, many substance abuse scientists anticipate that these policy changes may alter use patterns among adolescents due to its wider availability and a softening of beliefs about its potentially harmful consequences. Despite the possibility that these policies may increase the prevalence of use among adolescents, the effects of marijuana on neurodevelopment remain unclear, clouding arguments in favor of or opposition to these policies.

**Methods:**

The present prospective, longitudinal study was designed to isolate the neurodevelopmental consequences of marijuana use from its precursors during adolescence—a period of heightened vulnerability for both substance use and disrupted development due to environmental insults. Early adolescents who were substance-naïve at baseline (*N* = 529, aged 10–12) were recruited and tracked into adolescence when a subgroup initiated marijuana use during one of three subsequent waves of data collection, approximately 18 months apart.

**Results:**

Results suggest that marijuana use may be specifically related to a decline in verbal learning ability in the short term and in emotion recognition, attention, and inhibition in the longer-term.

**Discussion:**

These preliminary findings suggest that marijuana use has potential to adversely impact vulnerable neurodevelopmental processes during adolescence. Intensive additional investigation is recommended given that state-level policies regulating marijuana use and possession are rapidly shifting in the absence of good scientific information.

## Introduction

Adolescence is considered a transitional developmental period between childhood and adulthood during which significant changes in biological, social, and psychological functioning occur in the context of greater social demands and increasing autonomy (1). Due to adolescents’ heightened susceptibility to environmental influences, these changes pose challenges for developing teens who are attempting to navigate a rapidly evolving landscape of newfound independence, drives, and abilities. To do so successfully, ongoing maturation of higher order cognitive skills and emotion regulatory functions are relied upon. However, there is a disconnect between the phasic development of cognitive and emotional systems that would otherwise enable adaptive decision-making, impulse control, and self-regulation of behavior. The heuristic “dual systems model” suggests that the neural substrates of executive cognition (e.g., prefrontal cortex) are more easily overridden by a relatively more mature limbic system that undergirds emotional responses (2). Especially pronounced in the presence of peers, cognitive controls are not as effective in modulating emotions during adolescence as in adulthood (3), leading to somewhat higher rates of risky behaviors; a phenomenon characterized over the centuries as normative (4). However, youth with nonnormative maturational delays in prefrontal cognitive systems and their circuitry with lower regions are at heightened risk for a range of more serious risky behaviors, such as substance misuse (3).

In addition to increased risk for using abusable substances during this period, there is some evidence that these emergent systems may be more vulnerable to damage potentially caused by substance use (5). The effects of substance use may, thus, be observed in a maturational delay in those cognitive functions that have not yet reached their peak of maturity. Furthermore, in adolescents exhibiting a nonnormative lag in developing prefrontal regions, those “weaker” systems may be most adversely impacted by subsequent use. Studies have yet to fully establish whether and to what extent substance use during adolescence impedes development of various dimensions of executive cognitive functioning, with potential for enduring effects into adulthood. Also not well defined is the drug-specific nature of these impacts.

These questions are particularly salient when considering substances that are widely used in adolescence. Several national surveys consistently document that marijuana use substantially rises when children reach their adolescent years; adolescence is the stage when initiation most commonly occurs and marijuana is the most prevalent drug used, other than alcohol (6). Data from the Monitoring The Future (MTF) study concluding in 2019 affirms that rates of marijuana use rise sharply in adolescence, with 11.8% of the 8th graders, 28.8% of the 10th graders, and 35.7% of the 12th graders surveyed nationally reporting marijuana use in the past year (7). And more recently, both adolescents and young adults are reporting a historically even higher level of marijuana use (8, 9), perhaps due to decreased beliefs in the negative consequences of marijuana use due to legalization trends (10, 11).

Although these prevalence rates are alarming given the heightened vulnerability of the adolescent brain, there is a paucity of longitudinal efforts to isolate effects of marijuana on development, particularly across different levels of use and individual characteristics. The Adolescent Brain Cognitive Development (ABCD) study will eventually reveal both precursors and consequences of marijuana and other substance use in adolescents (12). Until then, there is an urgency to elucidate the impacts of marijuana use on neurodevelopment during this critical period of time in light of policy changes that are already directly influencing prevalence rates. Accurate and timely information is needed to ensure policies are grounded in science, and to educate parents, teachers, and adolescents about scientific findings and implications of policy change for their own decision-making.

The adolescent brain undergoes dramatic neural reorganization, including synaptic pruning and myelination (13), thereby facilitating greater differentiation across brain regions and increasing the speed of processing. This progression of brain remodeling occurs throughout adolescence into young adulthood, resulting in enhanced structural neural integration (14), which is integrally related to the fine-tuning of executive functioning, such as decision-making and emotion regulation (14–16). During remodeling, sensitivity to environmental stimuli is also heightened, as seen in augmented neural and physiological responses to negative peer interaction, mass media, and exposure to neighborhood influences (17, 18).

In similar fashion, the adolescent brain is more sensitive to the rewarding properties of abusable substances than adults (5, 19, 20), with potential for use to alter the course of structural and functional maturation (21). Repeated activation of the “reward” dopaminergic neural circuity occurs in response to substance use, sensitizing those neural systems and, in turn, reinforcing drug taking and seeking behaviors (22). The effects of marijuana on neural maturation are a focus of this research because it is most often initiated in adolescence and stimulates the dopaminergic reward system to achieve its desirable effects. Thus, a determination of whether and how tetrahydrocannabinol (THC) exposure, the primary active component of marijuana (23–25), impacts the developing brain is crucial. Studies suggest that THC alters the structure and function of the hippocampus and orbitofrontal cortex, potentially impairing memory, attention, and thinking and learning ability (26, 27). Additionally, similar to other abusable substances, adolescent exposure to THC sensitizes the brain to stimulation from various other drugs—known as cross-sensitization—heightening reward system responsivity and reinforcing further drug-taking (18).

The neural networks affected by THC subserve neurocognitive development; thus, marijuana-induced alterations in the functioning of those circuits are likely to be expressed in altered cognitive functioning, acutely and possibly sustainably. Chronic marijuana use has been linked later in life to aberrations in neural architecture that undergird aspects of neurocognition, including decreased white matter integrity and cortical thickness, associated with inattention and cognitive instability. This relationship is stronger with early initiation during adolescence and greater severity of use (14, 28). Heavy marijuana users also exhibit attenuated emotional responses to negative affective stimuli compared to healthy controls (29, 30) as well as impairments in memory and attention (18, 31, 32). And early initiators exhibit poorer executive cognitive functioning (33, 34). For example, early onset before age 16 is associated with a decline in verbal learning and verbal working memory compared to non-users (35, 36). There is some evidence that these adverse effects may be irreversible (18).

The evidence is not yet clear about whether these deficits are due to residual effects of active marijuana ingredients remaining in the system or potentially longstanding, direct neurotoxic effects of marijuana (37). Scott and his colleagues conducted a meta-analysis of studies that focused on heavy marijuana use in adolescents and young adults (38). Overall, results showed a small significant effect on cognitive functioning; however, that effect becomes insignificant when marijuana users were abstinent for longer than 72 h. A finer grained analysis by Hanson and her colleagues found an interesting pattern that suggests, after marijuana use is discontinued, some cognitive functions recover with time, while others appear to be more sustainably in deficit (35). Crean, Crane, and Mason reported a similar pattern where impaired executive functioning was found immediately after use, whereas in the long term, many of the residual effects diminished and functioning returned to baseline level (39). Very few of these studies included a substance-naïve baseline and many examined cognitive functioning only during acute administration conditions, negating the ability to ascertain whether deficits existed prior to use or were exacerbated by or entirely a consequence of use.

Whether adolescent marijuana use contributes to executive cognitive function and emotion recognition deficits requires prospective, longitudinal investigations, uniquely capable of delineating different dimensions of these processes and their developmental trends potentially impacted by marijuana use during adolescence. Also needed is research that accounts for the comorbid use of marijuana and other substances; few previous studies include other substance use as a covariate or an interaction term is used. Isolating the impact of marijuana from other substances is necessary to determine how it may alter the process of neurocognitive development. Policies can then be more appropriately and safely formulated based on the evidence and the public can be informed of any potential risks prior to the enactment of policy reforms.

This preliminary investigation analyzed data from the Longitudinal Study of Adolescent Marijuana Use and Neurodevelopment, funded by the National Institute on Drug Abuse (1R01DA022321-01A1; MPIs Drs. Diana Fishbein and Christopher Krebs), a prospective, longitudinal study designed to elucidate neurodevelopmental and psychosocial risk factors that predict marijuana initiation and escalation, and evaluate the impact of subsequent marijuana and other substance use on adolescent neurocognitive development. Benefitting from a substance-naïve baseline, the study was able to partial out the consequences of marijuana use on neurodevelopment from the precursors in early adolescence. Several tasks measuring executive cognitive function and emotion recognition were administered for a comprehensive examination of the specific dimensions predictive of and impacted by marijuana initiation and continuous use. Based on previous research, we anticipated that marijuana initiation and repeated use have different etiologies and may bi-directionally influence the development of executive function and emotional recognition (40–42). Therefore, we first hypothesized that youth who reported marijuana use in subsequent data collection waves would exhibit lower levels of functioning at baseline relative to non-users. Second, we expected marijuana use to exert negative impacts on neurodevelopment in both executive cognitive functioning and emotion recognition domains, controlling for baseline levels of functioning. As THC appears to exert its effects largely in the hippocampus and dorsolateral prefrontal cortex (43), we expected to find impairments in attention and working memory ability, leading to deficits in the ability to learn and perform complicated tasks.

## Materials and methods

### Participants

In 2018, adolescents (*N* = 529) were recruited from a working class, medium-sized city in northern Kentucky characterized by a high rate of early marijuana initiation compared to state and national rates (44). Administrators of local schools were contacted for access to information on enrolled students who were 10–12 years old (public domain information). Eligibility criteria included (a) willingness and ability to provide parental consent and youth assent; (b) English speaking; (c) not emotionally disturbed or severely learning disabled as reported by teachers or parents; and (d) have never consumed more than small amounts of alcohol (e.g., one standard drink) and no use of illicit substances by the time of the baseline interview. Study staff used a variety of strategies to recruit students, including: (i) posters and flyers distributed in schools; (ii) mailings to households with a study package that included a Superintendent’s endorsement letter and study brochure; and (iii) direct contact with custodial caregivers (44). Parents were compensated with cash and youth were given gift cards. This study was granted approval from the Institutional Review Boards (IRBs) of RTI International, University of Maryland School of Medicine, and The Pennsylvania State University. Data collection concluded at the end of 2013.

### Measures

Well-trained Master’s level research associates conducted separate sessions with caregiver and child within the household in a private location. Interviews were computer-assisted and sensitive content questions were asked using Audio Computer-Assisted Self-Interview (ACASI) technology. Survey measures were administered to both the parent or child and a battery of psychiatric and neurocognitive measures was administered to the child.

#### Substance use

A detailed survey of substance use was adapted from three large national surveys: the National Survey on Drug Use and Health (formerly known as the National Household Survey on Drug Abuse), the Monitoring The Future (MTF) Survey, and the Youth Risk Behavior Survey (YRBS) (44). Participants completed the survey in a private location in their homes and were repeatedly reminded that their responses were confidential and anonymous. Audio Computer-Assisted technology (ACASI) was used to increase accuracy of their responses (45). The range of substances gaged included marijuana, alcohol, tobacco, powder cocaine, crack, hallucinogens, heroin, inhalants, prescription pain relievers, Salvia, and stimulants (i.e., a range commonly abused amphetamines), with open-ended questions querying about any other substance use not listed. For each of them, ever use, past 30 days and cumulative days of use were collected to discern patterns of substance use overtime. A dummy variable representing “ever use” of all measured substances other than marijuana was created.

#### Functional domains of interest

All tasks are developmentally appropriate and have been validated in this age group and have shown minimal repeated measures effects (see references cited in each subsection).

##### Vigilance continuous performance test

The Vigilance Continuous Performance Test (CPT) is a measure of sustained attention and the ability to inhibit a prepotent impulse (46, 47). The screen presents a 2 × 2 matrix comprising two letters and two solid blocks. Participants are shown a sequence of letters on different positions in the matrix at a rate of 900 ms. Primary outcomes are the number of times the participant correctly responds to a nontarget letter, the number of misses and the number of incorrect responding.

##### Rey auditory-verbal learning test

The Rey Auditory-Verbal Learning Test (RAVLT) is a straightforward paper-and-pencil test which aims to measure the capacity of short-term memory and learning ability under proactive interference (48). The task starts with a list of 15 unrelated words at the rate of one word per second, followed by four additional trials. Participants are asked to remember what they see on the first trial and the fifth trail. Immediately and 30 min later (delayed recall), participants are given a story and asked to circle the words which appeared in each of the two trials. Number of correct recall and errors were used.

##### Motor restraint task

The Motor Restraint Task (MRT) measures inhibition of impulsive motor reactivity while executing a controlled slow motoric response (49). During this task, a narrow 108-degree circular arc is displayed across the screen. Participants are asked to trace the arc with stylus, and continuously move forward without going outside the lines, stopping or moving backward. The primary outcomes of the task are the time to traverse the arc and the time spent in the arc (number of stoppages).

##### Wheel of fortune

The Iowa Gambling Task (50–52) is a computerized measure of ability to develop a decision-making strategy based on previously learned information and sensitivity to consequences. The “Wheel of Fortune” (WoF) version (53, 54) was adapted to be developmentally appropriate for young adolescents. Players must develop a strategy to maximize gain, balancing between rewards and penalties; the disadvantageous strategy is the selection of large rewards with greater odds of losing those rewards. Outcome measures included percentage of risky selections, percentage of safe selections, and the risky/safe ratio.

##### Emotional stroop task

An Emotional Stroop Task assesses cognitive performance in the context of emotional stimuli (55). Respondents are presented with 45 words, 15 in each of positive, negative and control categories. The words are carefully chosen to represent positive and negative emotional states. Respondents are asked to state the color of the word while disregarding its content. Mean reaction times are calculated for each of the three categories. The differences in reaction times for positive and negative categories compared to the control category are primary outcome measures.

##### Facial recognition task

The Facial Recognition Task was developed at NIMH and uses facial pictures to measure the ability of emotion recognition (56, 57). Pictures of six emotional expressions are presented for participants to identify: happiness, sadness, anger, surprise, disgust, and fear. An additional category of neutral faces is also presented, which shows plain and alert faces. Participants are asked to recognize and label the emotion expressions in each of the seven categories. Number of correct identifications for each category and a total score were generated.

##### Trail making test

The Trail Making Test (TMT) provides information about speed for attention, mental flexibility, and executive functioning (58, 59). Fifteen circles containing numbers are randomly arranged on the screen. In trial A, respondents are asked to connect dots in an ascending pattern, and in trail B, they are asked to connect in the reversing order. The time spent in both trials was recorded.

### Data analysis strategy

All analyses were performed in R (version 3.6.1, 2019).

#### Exploratory factor analysis

An exploratory factor analysis (EFA) was conducted to assess the relationship and structure of the large set of measures included in this study. Using principal axis factoring with oblique rotation, EFA identified latent constructs underlying the measures and indicated dimensions of executive functioning and emotion recognition that clustered together. Eigenvalue and scree plot were used to determine the number of factors, and the “constructs” were interpreted based on the measures within each factor. A threshold of 0.4 was set for the minimum loading coefficient for items under each factor loading (60).

**Figure 1:**
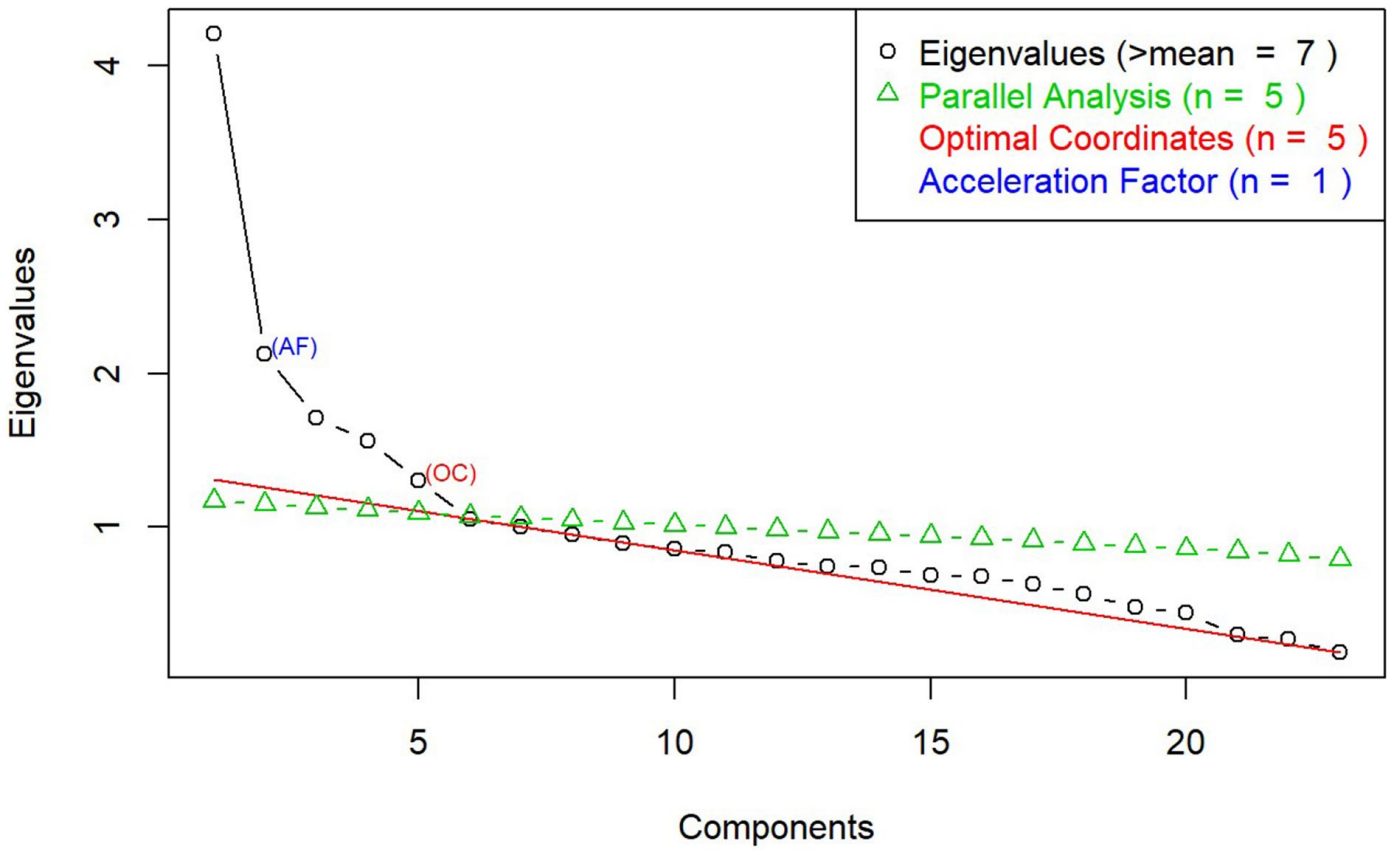
Scree plot from exploratory factor analysis on executive functioning and emotion recognition measures.

The EFA generated a seven-factor model solution with eigenvalues higher than 1. However, the scree plot suggested that a five-factor model was a better fit (see details in Figure 1). These five factors were labeled accordingly as: (1) Emotion Recognition (Facial Recognition Task); (2) Verbal Learning (RAVLT); (3) Mental Flexibility (MRT); (4) Emotion Repression (Emotional Stroop Task); and (5) Attention and Impulsivity (CPT).

#### Baseline differences in cognitive functioning

Exploratory regression analyses were performed to assess the relationship between the dichotomous measure of marijuana use at baseline and five factors from the EFA at baseline. Adolescents who later reported marijuana use were better able to accurately attribute emotion at baseline, compared to non-users over the course of this project.

#### Analysis of covariance

Analysis of covariance (ANCOVA) was used to examine whether cognitive performance reflected by each factor differs between the marijuana user group and non-user group by the end of wave 4 of data collection. Baseline differences between the two groups, with users exhibiting lower cognitive ability, might suggest a preexisting susceptibility to marijuana use in early adolescence (21). An ANCOVA was then conducted, controlling for baseline cognitive ability.

#### Growth modeling

The outcomes of interest included the dimensions of executive function and emotion recognition measured in this study. The primary analysis examined the relationship between marijuana use and level of executive functioning. An interaction term between marijuana and age was added to the model to test our hypothesis that there may be time varying effects on cognitive development for marijuana users. The model is specified as:


Level1:

$$\begin{array}{l} \mathrm{Facto}r_{ti}=\pi_{0i}+\pi_{1i}Age_{ti}+\pi_{2i}\mathrm{Marijuan}a_{ti}\\ \;+\pi_{3i}\mathrm{Age}\;X\;\mathrm{Marijuan}a_{ti}+e_{ti} \end{array}$$

Level2:

$$\pi_{0i}=\gamma_{00}+\gamma_{01}\;\mathrm{Incom}e_{i}+\gamma_{02}\mathrm{OtherSubstanc}e_{i}+\upsilon_{0i}$$

$$\pi_{1i}=\gamma_{10}+\upsilon_{1i}$$

$$\pi_{2i}=\gamma_{20}$$

$$\pi_{3i}=\gamma_{30}$$

#### Level of marijuana use

Previous research emphasizes the criticality of accurately measuring the propensity and severity of marijuana use (61). Therefore, marijuana use was coded in two ways: “ever use” and severity of use. For the ever use measure, participants who reported using marijuana at any wave were designated as users. For severity of use, the cumulative days of use by the time of assessment were coded into seven categories: 1 day, 2 days, 3–5 days, 6–9 days, 10–19 days, 20–39 days, and 40 or more days.

#### Covariates

All models included two covariates: the reported use of substances other than marijuana to isolate the effects of marijuana and household income, measured on an 11-point scale, ranges from less than $5,000 to $100,000–$149,999 per year. Previous studies have reported inconsistencies in the role of income. Some studies have found that marijuana use is negatively correlated with income level (62, 63), while others are contradictory, suggesting that higher household income is associated with higher rates of marijuana use (64, 65). Although comparisons between groups on household income at baseline were not significantly different, we included it in the model to avoid a possible confounding effect.

#### Missing data

Initial analyses included all participants with complete data for waves 1 and 2 (see below); full data for the cognitive tasks were required to render an accurate assessment. Fewer participants were included in the study beginning in wave 3 due to locating difficulties and budget constraints: 57.2% for wave 3 and 53.1% for wave 4. Chi-square tests showed no differences for sex or ethnicity between participants retained and participants lost to follow up for both waves 3 and 4. Thus, we used Multivariate Imputation by Chained Equation (MICE) which replaces missing values based on patterns in the overall dataset and their responses in the first two waves to compensate for their “loss.” MICE considers uncertainty in predicting missing values, reducing possible attrition bias, to provide a better prediction of developmental trajectories (66, 67).

## Results

### Characteristics of the population

Of the 529 participants recruited, 64 participants were excluded due to incomplete data in waves 1 and 2; therefore, 465 participants were included in the final analysis. Demographic characteristics of the full sample are presented in Table 1, including age, sex, ethnicity, and income. User status was determined based on whether participants reported using marijuana during wave 2, 3, or 4. The number of marijuana users was 148, while 317 were categorized as non-users. At baseline, marijuana users and non-users were similar in background characteristics, except for age; the user group was, on average, older at baseline (W = 20,600, *p* = 0.024) compared to the non-user group. Expectedly, as the prevalence rates of marijuana increases with age, an older age is associated with higher probability of having used marijuana by the end of the study; thus, no adjustment was needed. Group differences were examined using nonparametric statistics, given that they differed in size. Additionally, 137 of the 148 marijuana users indicated the number of days of “ever used” at wave 4: 1 day = 27; 2 days = 12; 3–5 days = 12; 6–9 days = 8; 10–19 = 10; 20–39 days = 10; and 40 or more days = 58. No significant differences in sex or ethnicity were found for any number of days of marijuana use.

**Table 1:** Baseline demographics by user status at wave 4.

Measure	Marijuana users (*N* = 148)	Non-user (*N* = 317)	*w* value	*p* value
Age			20,600	0.024^*^
10	34.4%	43.8%		
11	27.7%	28.1%		
12	37.2%	27.4%		
13^**^	0.7%	0.6%		
Sex			21,964	0.371
Male	47.3%	51.7%		
Female	52.7%	48.3%		
Ethnicity			22,582	0.778
White	49.3%	53.2%		
Black	37.0%	29.3%		
Other	13.7%	17.5%		
Household Income	5.21 (SD = 3.12)	5.63 (SD = 2.99)	25,575	0.114

^*^p ≤ 0.05; ^**^Children were enrolled from age 10–12, although a few became 13 by the time of testing.

### Baseline group differences in cognitive and emotion functioning

Table 2 presents results from the exploratory regression analyses. Emotion recognition was significantly different at baseline between the two groups (*t* = 0.23, *p* < 0.05), with adjustments for an age effect (68).

**Table 2:** Regression analysis of baseline differences in executive function and emotion recognition.

	Emotion recognition		Verbal learning		Mental flexibility		Emotion repression		Attention and impulsivity	
Measure	Estimate	SE	Estimate	SE	Estimate	SE	Estimate	SE	Estimate	SE
Intercept	−2.64^*^	0.65	−1.43	0.63	−0.98	0.53	0.27	0.68	2.28^*^	0.40
Age	0.19^*^	0.06	0.11	0.06	0.09	0.05	−0.01	0.06	−0.18^*^	0.04
Marijuana Use	0.23^*^	0.11	0.17	0.11	−0.11	0.09	−0.18	0.11	0.02	0.07

^*^p ≤ 0.05. (Marijuana) is the dichotomous variable measuring whether participants use marijuana by the end of wave 4.

### Functional group differences after initiation

One-way ANCOVAs were conducted to identify statistical differences between marijuana users and non-users in the level of executive cognitive function, controlling for other substance use, household income, and the function itself at baseline. Results are presented in Table 3. A significant effect of any marijuana use on verbal learning emerged [*F* (1,460) = 4.474, *p* < 0.05].

**Table 3:** ANCOVA comparison of five factors for Marijuana users and non-users.

Cases	Sum of squares	df	Mean square	*F*	*p*
*Difference in emotion recognition*					
Marijuana	1.175	1.000	1.175	1.972	0.161
Other substance	0.087	1.000	0.087	0.147	0.702
Income	0.591	1.000	0.591	0.991	0.320
Emotion recognition (Baseline)	63.799	1.000	63.799	107.041	< 0.001
Residual	274.172	460.000	0.596		
*Difference in verbal learning*					
Marijuana^*^	2.135	1.000	2.135	4.474	0.035
Other substance	0.465	1.000	0.465	0.974	0.324
Income	0.337	1.000	0.337	0.707	0.401
Verbal learning (Baseline)	78.004	1.000	78.004	163.496	< 0.001
Residual	219.467	460.000	0.447		
*Difference in mental flexibility*					
Marijuana	1.113	1.000	1.113	1.557	0.210
Other substance	0.302	1.000	0.302	0.427	0.514
Income	0.200	1.000	0.200	0.282	0.595
Mental Flexibility (Baseline)	23.558	1.000	23.558	33.321	<0.001
Residual	895.819	460.000	3.702		
*Difference in emotion repression*					
Marijuana	0.049	1.000	0.049	0.136	0.713
Other substance	0.629	1.000	0.629	1.758	0.186
Income	0.256	1.000	0.256	0.716	0.398
Emotion Repression (Baseline)	0.516	1.000	0.516	1.444	0.230
Residual	164.459	460.000	0.358		
*Difference in attention and impulsivity*					
Marijuana	0.041	1.000	0.041	0.109	0.742
Other substance	0.406	1.000	0.406	1.074	0.301
Income	1.464	1.000	1.464	3.876	0.050
Attention and Impulsivity (Baseline)	10.024	1.000	10.024	26.545	< 0.001
Residual	173.709	460.000	0.378		

^*^p ≤ 0.05. (Marijuana) is the dichotomous variable measuring whether participants use any marijuana by the end of wave 4. (Other substance) is the dichotomous variable measuring whether participants use any substance other than marijuana by the end of wave 4.

Additional one-way ANCOVAs were conducted to further explore whether days of use have differential effects on adolescent cognitive functioning (Table 4). We found a significant effect of days of marijuana use on emotion recognition [*F*(7,234) = 2.52, *p* < 0.05].

**Table 4:** ANCOVA comparison of five factors based on days of Marijuana use.

Cases	Sum of squares	df	Mean square	*F*	*p*
*Difference in emotion recognition*					
Marijuana^*^	6.766	7.000	0.967	2.515	0.016
Other substance	0.105	1.000	0.105	0.272	0.602
Income	0.050	1.000	0.050	0.131	0.718
Emotion recognition (Baseline)	31.295	1.000	31.295	81.449	< 0.001
Residual	89.911	234.000	0.384		
*Difference in verbal learning*					
Marijuana	8.095	7.000	1.156	1.882	0.073
Other substance	0.028	1.000	0.028	0.046	0.831
Income	0.014	1.000	0.014	0.023	0.880
Verbal learning (Baseline)	46.426	1.000	46.426	75.566	< 0.001
Residual	143.765	234.000	0.614		
*Difference in mental flexibility*					
Marijuana	4.703	7.000	0.672	0.730	0.646
Other substance	0.637	1.000	0.637	0.693	0.406
Income	0.541	1.000	0.541	0.588	0.444
Mental flexibility (Baseline)	7.869	1.000	7.869	8.554	0.004
Residual	215.262	234.000	0.920		
*Difference in emotion repression*					
Marijuana	1.705	7.000	0.244	0.576	0.775
Other substance	0.117	1.000	0.117	0.277	0.599
Income	0.132	1.000	0.132	0.311	0.577
Emotion repression (Baseline)	0.410	1.000	0.410	0.970	0.326
Residual	98.964	234.000	0.423		
*Difference in attention and impulsivity*					
Marijuana	2.197	1.000	0.314	0.705	0.667
Other substance	0.198	1.000	0.198	0.445	0.505
Income	1.195	1.000	1.195	2.684	0.103
Attention and impulsivity (Baseline)	3.859	1.000	3.859	8.672	0.004
Residual	104.131	234.000	0.445		

^*^p ≤ 0.05. (Marijuana) is the categorical variable measuring the cumulative days of marijuana use by the end of wave 4. (Other substance) is the dichotomous variable measuring whether participants use any substance other than marijuana by the end of wave 4.

### Developmental change in function in response to level of use

Table 5 presents results of growth models predicting cognitive and emotion functioning by adolescent marijuana use. The age variable was centered such that the zero-point represents the age of 10 which is the youngest age of participants when entering the study. Our five factors were each tested for both main effect of cumulative days of marijuana use and the interaction between age and cumulative days of marijuana use. Both the main effects of level of marijuana use and interaction effects of marijuana use and age were significantly predictive of Emotion Recognition (*t*_main_ = 0.64, *p* < 0.05; *t*_int_ = −0.05, *p* < 0.05) and Attention & Impulsivity (*t*_main_ = −0.45, *p* < 0.05; *t*_int_ = 0.03, *p* < 0.05).

**Table 5:** Results for conditional growth model.

	Emotion recognition		Verbal learning		Mental flexibility		Emotion repression		Attention and impulsivity	
	Estimate	SE	Estimate	SE	Estimate	SE	Estimate	SE	Estimate	SE
*Fixed effects*										
Initial level, γ_00_	−3.59^*^	0.244	−1.75^*^	0.25	−0.23	0.26	0.61	0.26	2.53^*^	0.20
Age, γ_10_	0.28^*^	0.019	0.12^*^	0.02	0.01	0.02	−0.04	0.02	−0.20^*^	0.02
Marijuana, γ_20_	0.64^*^	0.14	0.01	0.02	−0.09	0.18	0.05	0.16	−0.45^*^	0.12
Age by Marijuana, γ_30_	−0.05^*^	0.01	−0.01	0.01	0.01	0.01	0.00	0.01	0.03^*^	0.01
Income, γ_01_	0.02	0.01	0.03^*^	0.01	0.01	0.01	−0.01	0.01	−0.02^*^	0.01
Other Substance, γ_02_	0.00	0.01	0.08	0.08	−0.02	0.06	−0.03	0.06	0.01	0.05
*Random effects*										
Intercept, υ_0*i*_	2.93	1.71	0.30	0.55	1.00	1.00	5.98	2.45	3.76	1.94
Age, υ_1*i*_	0.01	0.08	0.00	0.01	0.01	0.12	0.03	0.18	0.03	0.16
Residual, e_ti_	0.43	0.65	0.50	0.71	0.63	0.79	0.65	0.81	0.26	0.51

^*^p ≤ 0.05; Marijuana is the categorical variable measuring the cumulative days of use by the end of wave 4. (Other Substance) is the dichotomous variable measuring whether participants use any substance other than marijuana by the end of wave 4.

## Discussion

The present study examined the relationship between level of neurocognitive functioning and marijuana use from a substance naïve baseline at age 10–12 until age 15–17. Researchers interested in a variety of problems extending from the use of marijuana have focused extensively on this period of development when adolescents are susceptible to use and may eventually misuse substances due to both environmental influences and neurodevelopmental vulnerability (2). Although most previous studies have not been able to isolate the neurocognitive consequences of marijuana due to the lack of a substance-naïve baseline, there is a growing number of investigations that will shed light on this outstanding question (69). At present, however, there are no longitudinal studies in the extant literature that employ growth models to ascertain both short-term and long-term effects of marijuana. In contrast with traditional analyses, growth modeling provides smooth trajectory estimation of developmental changes and is capable of incorporating time-varying predictors (70, 71). Further, the high concurrence between marijuana and other substance use demands adjustments for possible additive or synergistic effects of the use of more than one substance on developmental trajectories. Greater understanding of the relationship between marijuana use and neurocognitive development through adolescence can be achieved with the appropriate design features.

In the present investigation, EFA generated five latent constructs representing executive and emotion recognition dimensions—emotion recognition, verbal learning, mental flexibility, emotion repression, and attention and impulsivity—that were expected to show significant relative differences in their developmental progression between marijuana users and nonusers. Our purpose was to identify individual level functional indicators that are predictive of both marijuana initiation and consequences of use as an essential step in determining interventions and policies best suited to promote healthy neurodevelopmental trajectories.

To begin to address these outstanding scientific questions, our first hypothesis was that marijuana initiators will perform less well prior to onset on neurocognitive tasks compared to non-users. Instead, rather than weaker cognitive functioning prior to substance use initiation, we found only that adolescents with early onset of marijuana use were better able to accurately attribute emotion. Previous studies have reported that emotion recognition, particularly in response to negative emotional stimuli, is associated with marijuana initiation (44, 72). A parallel body of literature further suggests that history of child maltreatment, often accompanied by a higher sensitivity to negative emotions (73), has been associated with initiation of marijuana (74). Also potentially relevant is that adolescents who misuse marijuana have been found to report a higher level of aggression than non-initiators (75), which is further associated with over-attributing anger (76). In the present study, however, participants who began to use marijuana exhibited slightly higher scores for all emotions, with the total score reaching statistical significance. Further scrutiny is needed to evaluate the predictive value, significance, and mechanistic explanations for the relationship between emotion recognition and marijuana use.

Additional analyses tested the hypothesis that adolescents who initiate marijuana use will subsequently exhibit neurocognitive delays relative to adolescents who do not report use of marijuana. Because irreversible negative impacts on the developing brain and neurocognitive functioning may be attributable to early substance use (77), we anticipated that lower levels of neurocognitive functioning would be related to marijuana use. Partially consistent with expectations, the adolescent marijuana users in our sample exhibited slight deficits primarily in verbal learning ability. Compared to non-marijuana substance users and non-users, adolescent users recalled fewer words both immediately after learning and after a delay. This finding is supported by previous studies which suggest that the residual effect of marijuana is associated with reduced ability to memorize a word list (37).

In a third set of analyses to examine impacts of extent of marijuana use on development, two factors appeared to be affected. The growth model prediction suggested that repeated marijuana use may have hindered the development of attention and impulse control, beginning in late adolescence which is consistent with previous findings (78). With less attention to the environment, frequent marijuana users more often act without thinking and are less patient, potentially increasing unplanned risky behaviors including spontaneous substance use and violence (79). There is also ample evidence indicating that impulsivity is highly associated with vulnerability to addiction (80).

We further found a significant negative interaction effect between level of marijuana use and age on emotion recognition accuracy that may represent a long-term effect of frequent marijuana use. Emotion recognition is the focus of numerous previous studies to characterize marijuana users, however, they lack a substance naïve baseline (81–83). Nevertheless, based on those previous findings, we expected chronic marijuana users to exhibit difficulties in recognizing emotions, making the present findings intriguing. Although the marijuana user group performed better in the facial recognition task prior to initiation, this negative interaction over time suggests a decline in emotion recognition ability among frequent marijuana users that could become increasingly consequential in late adolescence into emerging adulthood. In effect, frequent marijuana users might experience difficulty in expressing empathy and attention, leading to peer relationship problems; accurately attributing facial expressions is important in interpersonal communications. There is also some evidence to suggest that marijuana users may consider consumption of substances as a substitute for friendship or a coping mechanism when relationship building is a challenge (84).

Overall, these findings implicate impaired acquisition and storage ability and heightened emotion recognition in adolescent marijuana users. However, unexpectedly, verbal learning ability was not identified in the growth modeling, suggesting that verbal learning is only influenced by marijuana in the short term. Lower levels of verbal learning ability, even when transient, may be expressed in poorer academic performance (85), potentially compounding risk for problematic substance use and other behavioral issues. It is possible that, with each use of marijuana, there is a short period of time when verbal learning ability is impaired, thereby affecting the ability to recall new words and discern connections between the words and their meaning (37), leading to difficulties in academic performance. The combination of academic challenges and heightened impulsivity during adolescence can have long-lasting consequences extending into adulthood, further increasing risk for ongoing or escalating use of marijuana and other substances.

These suppositions require further study to better understand how specific dimensions of EF predict and are impacted by initiation and patterns of use, and the potential for confounding effects when other substances are simultaneously used. Also of interest is the distinction between hot and cool EF, with the former involving emotional aspects of cognition (e.g., emotion regulation, reward-seeking, and impulsivity) and the latter involving neutral or decontextualized processes (e.g., verbal learning, cognitive flexibility, and working memory). In our study, both hot and cool EF were implicated in our measures of marijuana use at baseline and change over time. However, they played different roles. As aptly pointed out by Moriguchi and Phillips (86), these functions vary and begin to differentiate across development, showing different associations with behavioral patterns as well as neurobiological substrates. Understanding marijuana use precursors and consequences in this context is, thus, deemed an important line of inquiry.

There are a few implications of our findings for the development of intervention components that more specifically target these neurocognitive vulnerabilities to marijuana misuse and escalation. For example, programs to address trauma and bullying have potential to normalize attributions of negative emotions, thereby reducing aggressive behaviors and, in turn, may attenuate marijuana adverse effects on social interaction skills. Improving attention and reducing impulsivity are two additional skills that may offer some protection against using substances and experiencing their adverse effects in adolescence and beyond. Also, social emotional learning strategies build skills that tamp down on emotion reactivity, increase accuracy of emotion attribution in others and self, and improve classroom behavior and academic achievement (87). Information regarding neural circuits that undergird these abilities might further guide the selection of program components that act to strengthen precortical controls over emotion reactivity. Behavioral interventions that include focused meditation or other mindfulness practices and pharmacological approaches have been found to exert a beneficial effect at both the neural and behavioral levels (88, 89), improving the ability to cope with stress, strengthen cognitive control over emotional reactions, normalize emotion recognition, and calm aggressive tendencies, and may thus be useful in preventing marijuana use and dependence.

## Conclusion

There are several limitations in the present study. First, the sample size declined significantly across waves, particularly by wave 3. Relatedly, the reduced sample in waves 3 and 4 may have created selection bias, leading to a narrowing in the gap between developmental trajectories of executive function and emotion recognition of marijuana users and non-users in this study. Although we applied MICE strategy in treating missing data and to compensate for the decreased power of our analyses, the ability to detect true effects is compromised. Third, due to the high comorbidity between marijuana and other substances such as alcohol and tobacco, participants who only used marijuana without exposure to these other substances represented a relatively smaller number of marijuana-only users (20 out of 148). The small sample of marijuana-only users compromised the power of our analyses when controlling for other substance use. On the other hand, the use of other potentially more harmful substances (e.g., cocaine, opioids, and methamphetamine) was relatively rare. Additionally, discrepancies occurred when participants reported marijuana use in an earlier wave and then denied ever using in a later wave. We considered participants who reported ever use in any of the two waves marijuana users, even if use was denied in a subsequent wave.

Clearly, future research should focus on extending the span of time and number of participants to better depict the developmental trajectories of executive cognitive function and emotion recognition and ways in which marijuana use impacts developmental trajectories. Regardless, our results suggest a pathway for future studies to determine whether the decline we observed in a few of these processes among adolescent marijuana users holds. Furthermore, longitudinal studies from adolescence into early adulthood are needed to trace the widening or narrowing of the gap in functioning relative to typically developing control participants. Assessing the combined effects of multiple substance use will also enhance our understanding of how adolescent development is influenced specifically by marijuana. One possible direction is the application of a creative design (e.g., twin study with clean baseline) to disentangle the unique influence of marijuana, while another option is to focus on the polysubstance use effect rather than an individual drug.

During the past few years, legalization of recreational marijuana use has received an increasing amount of support from the public and policymakers. Nineteen states, two territories and the District of Columbia have legalized small amounts for recreational use as of May 2022 (90). Research suggests that, similar to alcohol and tobacco, legalization of marijuana may have negative effects on adolescent development; even though the sale to underage youth is illegal, such reforms can directly or indirectly influence use in young people (91). Concerns among substance abuse experts revolve largely around research consistently establishing that beliefs regarding negative consequences of the use of any given substance are directly and inversely related to risk for initiation among adolescents (10). Evidence is emerging that, indeed, there has been a clear decline in adolescents’ perception that marijuana use is hazardous in recent years (8). And in states where marijuana has been legalized, a precipitous rise in use among adolescents has been reported (11). Given these trends in perceptions that correspond with changes in marijuana laws, whether and how marijuana usage and neurodevelopment are affected would be an important consideration in policymaking. Policies based on evidence are inherently more effective in protecting public health. The same applies to marijuana laws, where well-informed policies will be more appropriately and safely formulated, and the public can be informed of any potential risks prior to the enactment of any policy reforms. In essence, understanding the impact of adolescent marijuana use on cognitive development is of great value to provide insights into both the benefits and harms of the legalization process. That information can inform the decision-making of policymakers enabling them to take into account the potential consequences of legalizing marijuana use for recreational purposes and ensure that appropriate safeguards are in place.

## Data availability statement

The raw data supporting the conclusions of this article will be made available by the authors, without undue reservation. Requests should be directed to dfishbein@unc.edu.

## Ethics statement

The studies involving human participants were reviewed and approved by RTI International. Written informed consent to participate in this study was provided by the participants’ legal guardian/next of kin.

## Author contributions

The parent study was conceived of and executed by DF, who also contributed to the conceptual framework for the present investigation and wrote and edited the manuscript. WR developed the hypotheses, selected variables for and conducted the analyses, contributed to interpretations, and co-wrote the paper. All authors contributed to the article and approved the submitted version.

## Funding

This investigation was supported by the National Institute on Drug Abuse (1R01DA022321-01A1).

## Conflict of interest

The authors declare that the research was conducted in the absence of any commercial or financial relationships that could be construed as a potential conflict of interest.

## Publisher’s note

All claims expressed in this article are solely those of the authors and do not necessarily represent those of their affiliated organizations, or those of the publisher, the editors and the reviewers. Any product that may be evaluated in this article, or claim that may be made by its manufacturer, is not guaranteed or endorsed by the publisher.
